# sST2 for Predicting Major Adverse Cardiovascular Events in STEMI Patients: A Prospective Cohort Study

**DOI:** 10.1155/crp/1443456

**Published:** 2026-11-10

**Authors:** Jinbo Wang, Wei Du, Xuesong He, Jinglin Cheng, Zeyan Liu

**Affiliations:** ^1^ Department of Emergency Internal Medicine Second Affiliated Hospital of Anhui Medical University Hefei China ahmu.edu.cn

**Keywords:** MACE, NT-proBNP, prediction, sST2, STEMI

## Abstract

**Background:**

Soluble suppression of tumorigenicity 2 (sST2) has emerged as a promising prognostic biomarker for cardiovascular conditions. Nevertheless, the prognostic value of sST2 following percutaneous coronary intervention (PCI) in patients with ST‐segment elevation myocardial infarction (STEMI) has been insufficiently studied. This study aimed to explore the association between sST2 levels and the incidence of major adverse cardiovascular events (MACE) in STEMI patients post‐PCI.

**Methods:**

This prospective, single‐center study enrolled 289 STEMI patients, who were randomly divided into training (*n* = 202) and validation (*n* = 87) cohorts at a 7:3 ratio. The primary endpoint was the occurrence of MACE within 6 months post‐STEMI. The relationships between sST2 concentrations and MACE were analyzed using Kaplan–Meier survival analysis, Cox proportional hazards regression models, and restricted cubic spline (RCS) plots. The predictive accuracy of sST2, NT‐proBNP, and their combined model was assessed using receiver operating characteristic (ROC) curves, concordance indices (C‐index), calibration plots, decision curve analysis (DCA), and net reclassification improvement (NRI). Subgroup analyses were conducted by stratifying patients based on age, gender, body mass index (BMI), and culprit vessel characteristics.

**Results:**

A total of 289 patients were included in the analysis. Kaplan–Meier survival curves in the training cohort demonstrated a higher cumulative incidence of MACE among patients with elevated sST2 levels. Multivariate Cox regression analysis confirmed sST2 as an independent predictor of MACE (HR = 1.012, 95% CI 1.006–1.018, *p* < 0.001). When compared to the traditional biomarker NT‐proBNP, sST2 displayed a comparable discriminative capability, with each biomarker offering unique strengths regarding accuracy and clinical usefulness. In the validation cohort, sST2 similarly exhibited acceptable predictive performance for MACE. Adding sST2 to the NT‐proBNP baseline model did not significantly improve the predictive performance for MACE in STEMI patients. Subgroup analyses did not reveal any significant interactions with sST2.

**Conclusion:**

In this study, sST2 was independently associated with MACE within 6 months post‐PCI in patients with STEMI and showed some discriminative ability when used alone. However, adding it to NT‐proBNP did not significantly improve risk prediction, and its incremental prognostic value and clinical utility remain limited and uncertain.

## 1. Introduction

Acute ST‐segment elevation myocardial infarction (STEMI) is caused by acute complete coronary artery occlusion due to secondary thrombosis from unstable plaques. This occlusion leads to myocardial ischemia and hypoxia, resulting in myocardial injury and necrosis [1]. Although reperfusion therapy can promptly save the lives of STEMI patients [2], some patients still experience major adverse cardiovascular events (MACE) after percutaneous coronary intervention (PCI), reducing their quality of life and increasing their risk of death [3]. Identifying possible postoperative MACE in STEMI patients has become a significant research area. This identification is clinically important for improving patient prognosis, guiding long‐term follow‐up of high‐risk individuals, and optimizing medical resource allocation.

Changes in myocardial structure, morphology, and function after infarction represent critical prognostic factors and intervention points. Studies have indicated that soluble suppression of tumorigenicity 2 (sST2) is a potential target for ventricular remodeling [4, 5]. Since the discovery of the ST2 gene in 1989, sST2 and ST2L have been identified as the two primary subtypes. These subtypes exert different biological effects depending on molecular structure and expression site [6]. As a member of the interleukin‐1 receptor family, sST2 is upregulated in cardiomyocytes and cardiac fibroblasts under mechanical stress or inflammatory stimulation and subsequently released into circulation. By competitively inhibiting the IL‐33/ST2 signaling pathway, sST2 blocks or reverses protective effects, such as inhibition of myocardial fibrosis, cardiomyocyte hypertrophy, and apoptosis. Therefore, sST2 has recently attracted significant attention as a cardiovascular biomarker [7]. Animal experiments have shown that compared to ST2‐deficient mice, mice expressing the ST2 gene exhibited reduced myocardial hypertrophy and fibrosis [8]. Clinical studies indicate that elevated sST2 levels correlate not only with overall mortality but also with MACE in acute myocardial infarction patients [9]. Moreover, sST2 reportedly promotes atherosclerosis and is closely associated with the formation, progression, and severity of unstable plaques [10, 11]. A recent multicenter study demonstrated that an integrated index derived from serum biomarkers (the BAB index) was validated in a cohort of 8002 STEMI patients and exhibited robust predictive performance for both short‐term and long‐term mortality [12]. The pursuit of novel biomarkers continues to evolve, and the association between elevated sST2 levels and adverse outcomes in STEMI patients warrants further investigation.

Currently, extensive research has shown that sST2 is significantly involved in predicting outcomes in cardiovascular diseases, particularly heart failure (HF). Elevated sST2 concentrations correlate with increased hospital readmissions and cardiovascular‐related mortality in HF patients [13, 14] and can be used for risk stratification in patients with HF [15]. However, the prognostic significance of sST2 in patients with STEMI is still underexplored. Given the impact of sST2 on cardiomyocyte function, it is plausible that sST2 could serve as a valuable prognostic biomarker in STEMI patients, warranting further investigation. This study aimed to prospectively examine the predictive capacity of sST2 in STEMI patients, comparing it against established biomarkers. The objective was to more accurately identify individuals likely to experience adverse outcomes, enabling patients to benefit more from enhanced monitoring and optimized treatment.

## 2. Methods

### 2.1. Research Subjects

From November 2023 to February 2025, 289 eligible patients were enrolled from the Second Affiliated Hospital of Anhui Medical University according to predefined inclusion and exclusion criteria (Figure 1).

**FIGURE 1:**
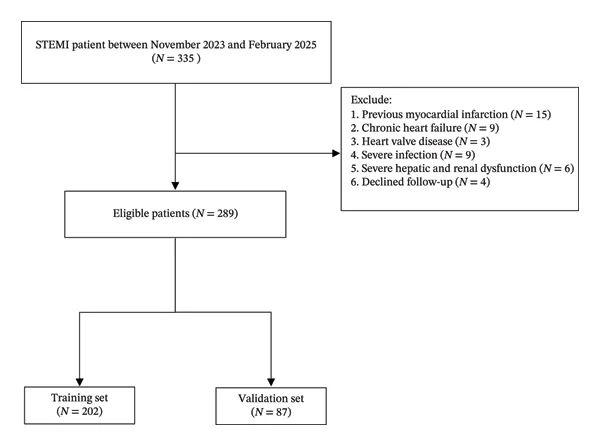
Study flow chart.

Inclusion criteria encompassed: (1) a confirmed diagnosis of STEMI following the 2017 European Society of Cardiology (ESC) guidelines for acute myocardial infarction with ST‐segment elevation [3]; (2) successful performance of PCI within 90 min of admission.

Exclusion criteria: (1) previous myocardial infarction; (2) heart valve disease, dilated cardiomyopathy, or hypertrophic cardiomyopathy; (3) severe liver or kidney dysfunction; (4) serious infections, autoimmune diseases, or malignancies; (5) refusal to participate in follow‐up.

The sample size calculation was based on the postoperative MACE incidence reported in a previous study by Huang et al. [16], in which the MACE rate was 8% in the control group and 28.8% in the experimental group. We applied the standard sample size formula for cohort studies, with a two‐sided significance level (*α*) of 0.05 and a power (1‐*β*) of 0.90. Considering a potential dropout rate of 10%, the calculated sample size was inflated by 10% and then rounded up, resulting in a minimum required sample size of 160 patients. To further ensure the robustness and reliability of the study conclusions, the final enrolled sample size was increased beyond this minimum requirement. The study adhered strictly to the Declaration of Helsinki principles, received ethical approval from the Ethics Committee of the Second Affiliated Hospital of Anhui Medical University (Approval No. YX2022‐001), and written informed consent was provided by all participants or their legal representatives.

### 2.2. Data Collection

Data collection included: (1) demographic characteristics: gender, age, body mass index (BMI), and medical history (including history of hypertension, diabetes, smoking, and alcohol consumption); (2) serological parameters obtained 24‐h post‐PCI, including C‐reactive protein (CRP), N‐terminal pro‐brain natriuretic peptide (NT‐proBNP), high‐sensitivity cardiac troponin I (Hs‐CTnI), myoglobin (MYO), creatine kinase–myocardial band (CK–MB), D‐dimer, glycosylated hemoglobin (HbA1C), total cholesterol (TC), triglycerides (TG), low‐density lipoprotein cholesterol (LDL‐C), uric acid (UA), and creatinine (Cr); (3) clinical data, including Killip class on admission, time of symptom onset, door‐to‐wire (D2W) time, culprit vessel, postprocedural TIMI flow grade, and SYNTAX score; (4) imaging parameters obtained on the third‐day post‐PCI by echocardiography, including left ventricular end‐diastolic volume (LVEDV), left ventricular end‐systolic volume (LVESV), left ventricular ejection fraction (LVEF), and interventricular septal thickness (IVS); (5) medication use: ACEI/ARB/ARNI, beta‐blockers, sodium–glucose cotransporter 2 inhibitors (SGLT2i), and mineralocorticoid receptor antagonists (MRA); (6) quantification of sST2: Peripheral venous blood samples (2 mL) were collected from all patients 24‐h post‐PCI and analyzed by enzyme‐linked immunosorbent assay (ELISA) using a commercial kit (Beijing Leadman Biochemistry Co., Ltd., Lot No. 24L25; detection range: 1–300 ng/mL). The intra‐assay coefficient of variation was < 10%.

### 2.3. Follow‐Up and Grouping

Patients were systematically followed every month after discharge for 6 months through standardized telephone interviews and outpatient visits. Throughout the follow‐up period, occurrences of MACE were meticulously documented, subsequently categorizing patients into groups based on the presence or absence of MACE. MACE included the following: (1) HF, diagnosed according to the 2022 AHA/ACC/HFSA HF management guidelines [13]; (2) recurrent myocardial infarction, defined as myocardial infarction after discharge due to restenosis or new‐onset coronary atherosclerosis; (3) new ischemic or hemorrhagic cerebrovascular events; (4) all‐cause mortality. Two independent experts determined all endpoint events using a blinded method. Loss to follow‐up was strictly minimized. All patients lost to follow‐up were treated as censored, meaning that their survival status and follow‐up duration at the last available visit were included as censored data in the analysis.

### 2.4. Statistical Analysis

Statistical analyses were executed using SPSS Version 27.0 and R Version 4.5.1, incorporating R packages such as survival, survminer, rms, timeROC, and dcurves. Continuous variables were reported as mean ± standard deviation (SD) and analyzed via independent‐samples *t*‐tests or expressed as medians with interquartile ranges, utilizing the Mann–Whitney *U* test for comparison. Categorical variables were described in frequencies (%) and analyzed using chi‐square or Fisher’s exact tests according to their distributional characteristics. A *p* value < 0.05 indicated statistical significance.

The patient cohort was stratified by MACE status and randomly divided into a training set and an internal validation set at a 7:3 ratio, so that the event rate was preserved in both sets. In the training set, patients were stratified into high and low sST2 groups according to the median sST2 level. Kaplan–Meier survival curves were used to compare survival rates between the two groups, and the log‐rank test was used to assess differences between the two survival curves. Restricted cubic spline (RCS) analysis was performed using R software to examine the dose–response relationship between sST2 and MACE. Univariate Cox regression analysis was first performed to screen for statistically significant variables. Based on the univariate analysis results, clinical relevance, and evidence from previous literature, appropriate variables were selected and entered into multivariate Cox regression analysis to identify independent risk factors for MACE. The proportional hazards (PH) assumption of the Cox model was assessed using Schoenfeld residual tests. In addition, we separately compared the predictive performance of sST2, NT‐proBNP, and their combined model in the training and validation sets. Evaluation methods included receiver operating characteristic (ROC) curves, concordance index (C‐index), calibration curves, decision curve analysis (DCA), and net reclassification improvement (NRI). Subgroup analyses were performed to evaluate the effect of sST2 across four subgroups based on age, gender, BMI, and culprit vessel, and interaction tests were used to assess the statistical significance of differences. Component‐specific analyses of the study endpoints were performed using descriptive methods. Event rates (per 100 person‐years) and 95% confidence intervals (CIs) were calculated for each component in the high and low sST2 groups. With the absolute rate difference of the total composite endpoint as the denominator, the contribution proportion of each component to the total rate difference was calculated.

## 3. Results

### 3.1. Baseline Characteristics of Patients

This analysis encompassed 289 patients, predominantly male (87.1%), with a mean age of 56.78 ± 13.42 years. Of the 202 patients in the training cohort, follow‐up was incomplete for six individuals, and 43 experienced MACE. These events included HF (*n* = 24), recurrent myocardial infarction (*n* = 11), mortality (*n* = 5), and stroke (*n* = 3). The validation cohort comprised 87 patients, two of whom were lost during follow‐up, and 18 who developed MACE. Baseline demographic and clinical characteristics were comparable between the two cohorts (Table 1).

**TABLE 1:** Balance test of baseline data between training set and validation set.

Variables	Total (*n* = 289)	Training (*n* = 202)	Validation (*n* = 87)	*p*
sST2, Median (Q1, Q3), ng/mL	38.85 (19.80–65.30)	38.59 (18.94–66.54)	40.30 (24.20–62.30)	0.478
Age, mean ± SD, years	56.78 ± 13.42	56.68 ± 13.58	57.01 ± 13.12	0.847
Gender, *n* (%)				0.329
Male	248 (87.1)	176 (87.1)	72 (82.8)	
Female	41 (12.9)	26 (12.9)	15 (17.2)	
BMI, *n* (%)				0.538
< 18.5 kg/m^2^	7 (2.4)	4 (2.0)	3 (3.4)	
18.5–2 4 kg/m^2^	152 (52.6)	102 (50.5)	50 (57.5)	
24–28 kg/m^2^	98 (33.9)	72 (35.6)	26 (29.9)	
≥ 28 kg/m^2^	32 (11.1)	24 (11.9)	8 (9.2)	
Killip, *n* (%)				0.810
Killip I	252 (87.2)	174 (86.1)	78 (89.7)	
Killip II	15 (5.2)	11 (5.4)	4 (4.6)	
Killip III	12 (4.1)	10 (5.0)	2 (2.3)	
Killip IV	10 (3.5)	7 (3.5)	3 (3.4)	
Hypertension history, *n* (%)				0.711
No	105 (36.3)	72 (35.6)	33 (37.9)	
Yes	184 (63.7)	130 (64.4)	54 (62.1)	
Diabetes history, *n* (%)				0.056
No	199 (68.9)	146 (72.3)	53 (60.9)	
Yes	90 (31.1)	56 (27.7)	34 (39.1)	
Smoking history, *n* (%)				0.107
No	84 (29.1)	53 (26.2)	31 (35.6)	
Yes	205 (70.9)	149 (73.8)	56 (64.4)	
Drinking history, *n* (%)				0.121
No	123 (42.6)	80 (39.6)	43 (49.4)	
Yes	166 (57.4)	122 (60.4)	44 (50.6)	
CRP, median (Q1, Q3), mg/L	2.80 (1.30–6.70)	2.90 (1.3–6.70)	2.70 (1.20–6.10)	0.616
NT‐proBNP, median (Q1, Q3), pg/mL	1060.00 (490.00–2010.00)	969.00 (459.00–1885.00)	1192.00 (573.00–2354.00)	0.240
Hs‐CTnI, *n* (%)				0.233
≤ 26,208.0 pg/mL	135 (46.7)	99 (49.0)	36 (41.4)	
> 26,208.0 pg/mL	154 (53.3)	103 (51.0)	51 (58.6)	
MYO, median (Q1, Q3), ng/mL	85.00 (50.20–243.70)	84.50 (48.50–245.40)	88.20 (50.70–238.00)	0.815
CK–MB, median (Q1, Q3), U/L	99.00 (57.00–228.00)	96.00 (55.30–237.00)	106.00 (65.00–219.00)	0.841
HB1AC, median (Q1, Q3), %	5.90 (5.60–6.90)	5.90 (5.60–6.50)	6.10 (5.70–7.40)	0.081
TC, mean ± SD, mmol/L	4.87 ± 0.97	4.91 ± 0.96	4.76 ± 0.98	0.224
TG, median (Q1, Q3), mmol/L	1.52 (1.01–2.28)	1.51 (1.00–2.22)	1.53 (1.03–2.43)	0.695
LDL‐C, mean ± SD, mmol/L	3.26 ± 0.92	3.32 ± 0.91	3.14 ± 0.93	0.143
D‐dimer, median (Q1, Q3), mg/L	0.31 (0.19–0.60)	0.31 (0.19–0.58)	0.31 (0.20–0.63)	0.531
UA, mean ± SD, umol/L	358.19 ± 79.41	359.70 ± 75.92	355.85 ± 87.37	0.722
Cr, median (Q1, Q3), umol/L	74.00 (62.00–86.00)	74.00 (63.00–85.00)	74.00 (61.00–86.00)	0.813
Onset time, median (Q1, Q3), hour	4.00 (2.00–9.00)	4.00 (2.00–9.00)	4.00 (1.50–9.37)	0.621
D‐to‐W time, median (Q1, Q3), min	64.00 (52.00–76.00)	63.00 (51.00–77.00)	64.00 (55.00–73.00)	0.707
Culprit artery, *n* (%)				0.675
LAD	148 (51.2)	100 (49.5)	48 (55.2)	
LCX	33 (11.4)	24 (11.9)	9 (10.3)	
RCA	108 (37.4)	78 (38.6)	30 (34.5)	
TIMI classification III, *n* (%)				0.495
No	10 (3.5)	6 (3.0)	4 (4.6)	
Yes	279 (96.5)	196 (97.0)	83 (95.4)	
SYNTAX score, median (Q1,Q3)	16.50 (12.50–19.50)	16.00 (12.00–19.50)	17.50 (13.00–20.00)	0.146
LVEDV, median (Q1, Q3), ml	97.00 (83.00–113.00)	96.50 (83.00–113.00)	97.00 (80.00–115.00)	0.854
LVEF, median (Q1, Q3), %	59.00 (50.00–62.00)	58.00 (51.00–62.00)	60.00 (48.00–62.00)	0.708
IVS, median (Q1, Q3), mm	10.00 (10.00–12.00)	10.50 (9.00–12.00)	10.00 (10.00–12.00)	0.837
ACEI/ARB/ARNI, *n* (%)				0.730
No	66 (22.8)	45 (22.3)	21 (24.1)	
Yes	223 (77.2)	157 (77.7)	66 (75.9)	
Beta‐blocker, *n* (%)				0.669
No	78 (27.0)	56 (27.7)	22 (25.3)	
Yes	211 (73.0)	146 (72.3)	65 (74.7)	
SGLT2i, *n* (%)				0.334
No	175 (60.6)	126 (62.4)	49 (56.3)	
Yes	114 (39.4)	76 (37.6)	38 (43.7)	
MRA, *n* (%)				0.547
No	233 (80.6)	161 (79.7)	72 (82.8)	
Yes	56 (19.4)	41 (20.3)	15 (17.2)	

*Note:* Hs‐cTnI, high‐sensitivity cardiac troponin I; MYO, myoglobin; HbA1c, glycosylated hemoglobin; TG, triglycerides; Cr, creatinine; IVS, interventricular septal thickness; SGLT2i, sodium–glucose cotransporter 2 inhibitors.

Abbreviations: ACEI, angiotensin‐converting enzyme inhibitor; BMI, body mass index; CK–MB, creatine kinase–myocardial band; CRP, C‐reactive protein; D‐to‐W, door to wire; LDL‐C, low‐density lipoprotein cholesterol; LVEF, left ventricular ejection fraction; LVEDV, left ventricular end‐diastolic volume; LVESV, left ventricular end‐systolic volume; MRA, mineralocorticoid receptor antagonists; NT‐proBNP, N‐terminal pro‐brain natriuretic peptide; sST2, soluble suppression of tumorigenicity 2; TC, total cholesterol; TIMI, thrombolysis in myocardial infarction; UA, uric acid.

### 3.2. sST2 Grouping

In the training cohort, patients were categorized into high and low sST2 groups according to the median sST2 level of 38.59 ng/mL. Kaplan–Meier survival analysis (Figure 2) indicated significantly increased cumulative MACE incidence in patients with high sST2 concentrations (*p* < 0.001).

**FIGURE 2:**
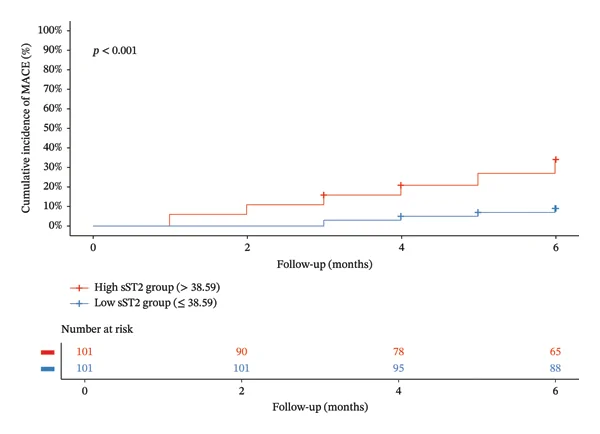
The cumulative incidence of MACE between the two groups.

### 3.3. Risk Factors for MACE

In univariate Cox regression analysis (Table 2), age, Killip class, CRP, NT‐proBNP, SYNTAX score, LVEF, SGLT2i, MRA, and sST2 emerged as significant risk factors for MACE. The multivariate Cox regression analysis (Table 3) included 10 covariates, with an events per variable (EPV) of 4.3. Multivariate analysis showed that sST2 (HR = 1.012, 95% CI: 1.006–1.018, *p* < 0.001) remained an independent risk factor for MACE. Sensitivity analysis using Firth’s penalized Cox regression model showed that sST2 (HR = 1.011, 95% CI: 1.005–1.017, *p* < 0.001) yielded results consistent with those of the standard Cox model, indicating that the main results were robust. The PH assumption was assessed using Schoenfeld residual tests; the global *p* value was 0.52, and the Schoenfeld residual test *p* values for all covariates were > 0.05. Furthermore, RCS analysis showed no significant nonlinearity between sST2 and MACE risk (Figure 3, *p* for nonlinearity = 0.117).

**TABLE 2:** Univariable and multivariable Cox regression analysis for MACE.

Variables	Univariable analysis		Multivariable analysis	
	HR (95% CI)	*p*	HR (95% CI)	*p*
sST2 (ng/mL)	1.013 (1.008–1.018)	**< 0.001**	1.012 (1.006–1.018)	**< 0.001**
Age (years)	1.057 (1.033–1.081)	**< 0.001**	1.054 (1.028–1.082)	**< 0.001**
Gender (female vs male)	0.573 (0.266–1.234)	0.155		
BMI (18.5–24.0 vs. < 18.5 kg/m2)	0.576 (0.077–4.289)	0.591		
BMI (24.0–28.0 vs. < 18.5 kg/m2)	0.622 (0.082–4.693)	0.645		
BMI (≥ 28.0 vs. < 18.5 kg/m2)	0.572 (0.067–4.897)	0.610		
Killip (I vs. non‐I)	0.448 (0.221–0.909)	**0.026**	0.592 (0.261–1.342)	0.209
Hypertension history (yes vs no)	1.948 (0.960–3.952)	0.065		
Diabetes history (yes vs no)	1.679 (0.905–3.116)	0.101		
Smoking history (yes vs no)	0.647 (0.346–1.212)	0.174		
Drinking history (yes vs no)	0.565 (0.311–1.028)	0.062		
CRP (mg/L)	1.040 (1.021–1.060)	**< 0.001**		
NT‐proBNP (> 969 vs. ≤ 969 pg/mL)	3.401 (1.714–6.751)	**< 0.001**	0.850 (0.368–1.968)	0.705
Hs‐CTnI (26,208.0 > vs. ≤ 26,208.0 pg/mL)	1.720 (0.927–3.192)	0.086	0.913 (0.451–1.846)	0.799
Myo (84.5 > vs. ≤ 84.5 ng/mL)	1.458 (0.796–2.673)	0.222		
CK–MB (96.0 > vs. ≤ 96.0 U/L)	0.970 (0.534–1.764)	0.921		
HbA1C (%)	1.174 (0.962–1.433)	0.115		
TC (mmol/L)	1.222 (0.899–1.661)	0.200		
TG (mmol/L)	0.730 (0.530–1.006)	0.054		
LDL‐C (mmol/L)	1.196 (0.857–1.669)	0.292		
D‐Dimer (mg/L)	1.236 (0.972–1.571)	0.083		
UA (umol/L)	1.003 (0.999–1.006)	0.210		
Cr (umol/L)	1.007 (0.992–1.023)	0.361	0.988 (0.972–1.005)	0.175
Onset time (hour)	1.022 (0.995–1.050)	0.118		
Culprit artery (LAD vs. non‐LAD)	1.307 (0.716–2.387)	0.383		
TIMI classification (III vs. non‐III)	1.436 (0.198–10.433)	0.721		
SYNTAX Score	1.140 (1.080–1.205)	**< 0.001**	1.091 (1.007–1.181)	**0.033**
LVEDV (mL)	1.007 (0.991–1.023)	0.392		
LVEF (%)	0.913 (0.885–0.943)	**<** **0.001**	0.920 (0.876–0.966)	**<** **0.001**
IVS (mm)	1.203 (0.995–1.454)	0.056		
ACEI/ARB/ARNI (yes vs. no)	0.926 (0.456–1.879)	0.832		
Beta‐blocker (yes vs. no)	1.240 (0.611–2.516)	0.552		
SGLT2i (yes vs. no)	2.670 (1.456–4.896)	**<** **0.001**	0.810 (0.380–1.726)	0.585
MRA (yes vs. no)	2.292 (1.224–4.292)	**0.010**	0.489 (0.209–1.149)	0.101

*Note:* Hs‐cTnI, high‐sensitivity cardiac troponin I; MYO, myoglobin; HbA1c, glycosylated hemoglobin; Cr, creatinine; SGLT2i, sodium–glucose cotransporter 2 inhibitors. Bold values represent *p* values less than 0.05.

Abbreviations: ACEI, angiotensin‐converting enzyme inhibitor; BMI, body mass index; CK–MB, creatine kinase–myocardial band; CRP, C‐reactive protein; D‐to‐W, door to wire; IVS, interventricular septal thickness; LDL‐C, low‐density lipoprotein cholesterol; LVEDV, left ventricular end‐diastolic volume; LVEF, left ventricular ejection fraction; MRA, mineralocorticoid receptor antagonists; NT‐proBNP, N‐terminal pro‐brain natriuretic peptide; sST2, soluble suppression of tumorigenicity 2; TC, total cholesterol; TG, triglycerides; TIMI, thrombolysis in myocardial infarction; UA, uric acid.

**TABLE 3:** Comparison of prognostic value of the different models at follow‐up time.

Model	Month	Training set	Validation set
		AUC (95% CI)	AUC (95% CI)
sST2	3	0.725 (0.606–0.844)	0.697 (0.526–0.868)
	6	0.727 (0.634–0.820)	0.725 (0.582–0.868

NT‐proBNP	3	0.745 (0.641–0.849)	0.706 (0.532–0.880)
	6	0.731 (0.648–0.814)	0.745 (0.620–0.870)

sST2 + NT‐proBNP	3	0.785 (0.706–0.864)	0.774 (0.617–0.931)
	6	0.799 (0.727–0.871)	0.789 (0.664–0.914)

**FIGURE 3:**
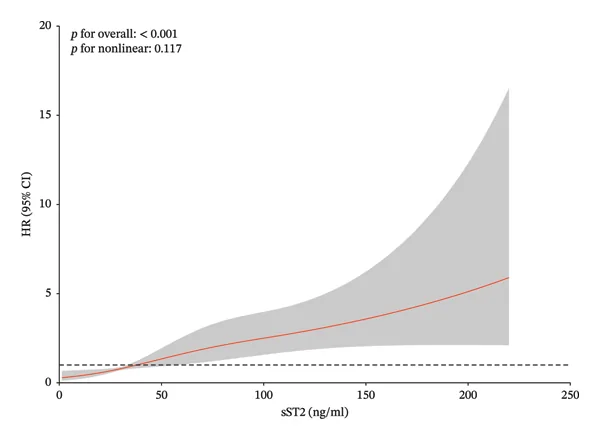
The dose–response relationship between sST2 and MACE.

### 3.4. Predictive Performance in the Training Cohort

In the training cohort, ROC analysis (Figure 4A,B) indicated no significant differences between sST2 and NT‐proBNP in discriminative power at both 3 and 6 months (AUC: 0.725 vs. 0.745, *p* > 0.05 by DeLong test at 3 months; AUC: 0.727 vs. 0.731, *p* > 0.05 by DeLong test at 6 months; Table 3). The C‐index similarly did not differ significantly between sST2 and NT‐proBNP (C‐index: 0.706 vs. 0.708, *p* > 0.05). Calibration curves (Figure 5A,B) displayed the predictive accuracy of sST2 and NT‐proBNP at different time points. DCA (Figure 5C,D) showed that at lower threshold ranges, NT‐proBNP and sST2 each had advantages, but NT‐proBNP performed better within certain thresholds (3 months: 0.15–0.35; 6 months: 0.3–0.65).

**FIGURE 4:**
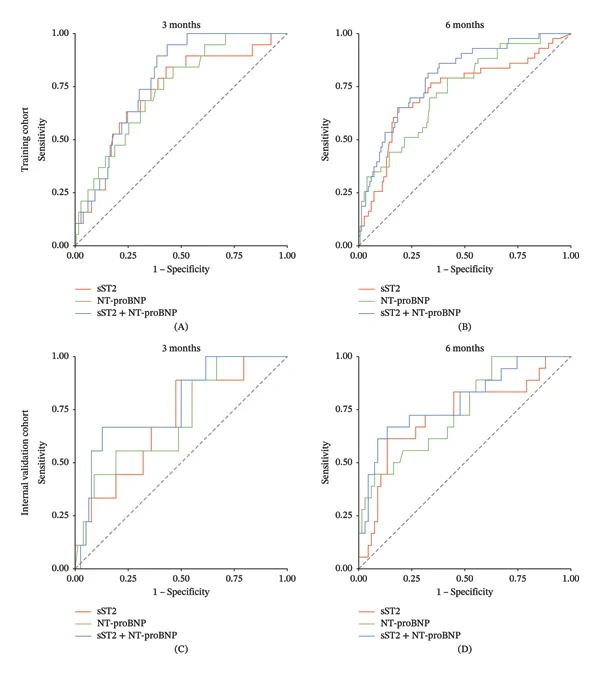
ROC curve for the prediction of MACE by sST2 and NT‐proBNP at follow‐up time, training cohort (A, B), internal validation cohort (C, D).

**FIGURE 5:**
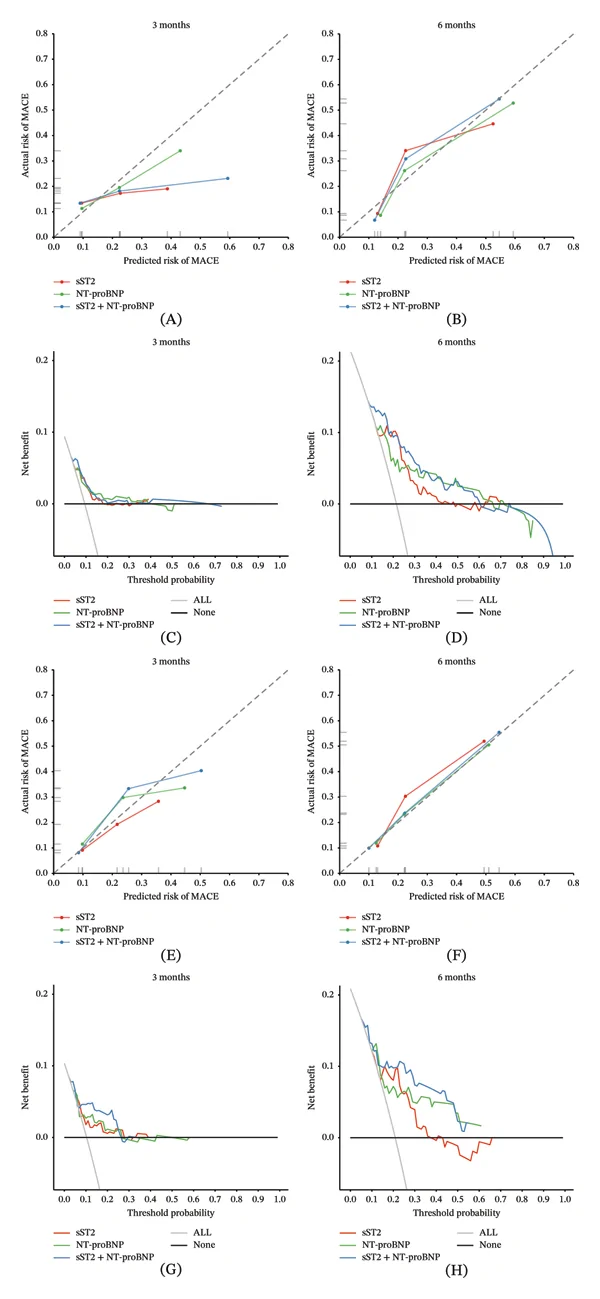
Calibration curve and DCA for the prediction of MACE by sST2 and NT‐proBNP at follow‐up time, training cohort (A–D), internal validation cohort (E–H).

### 3.5. Predictive Performance in the Internal Validation Cohort

ROC analysis in the internal validation cohort (Figure 4C,D) also revealed no statistically significant differences in predictive capacity between sST2 and NT‐proBNP at 3 and 6 months (AUC: 0.697 vs. 0.706, *p* > 0.05 by DeLong test at 3 months; AUC:0.725 vs. 0.745, *p* > 0.05 by DeLong test at 6 months; Table 3). The C‐index corroborated these findings, showing no notable differences between sST2 and NT‐proBNP (C‐index: 0.706 vs. 0.717, *p* > 0.05). Calibration curves (Figure 5E,F) illustrated the predictive accuracy of both biomarkers at different time points. DCA (Figure 5G,H) showed varying clinical net benefits of NT‐proBNP and sST2 at different threshold ranges.

### 3.6. Predictive Performance of sST2 Combined With NT‐proBNP

In the training cohort, after adding sST2 to the baseline model containing NT‐proBNP, the ROC analysis showed no significant difference in discriminative power between the combined model and NT‐proBNP at 3 and 6 months (AUC: 0.785 vs. 0.745, *p* > 0.05 by DeLong test at 3 months; AUC: 0.799 vs. 0.731, *p* > 0.05 by DeLong test at 6 months; Figure 4A,B, Table 3). The C‐index increased from 0.708 to 0.769, but this improvement did not reach statistical significance (*p* > 0.05). The NRI was 0.098 (*p* > 0.05), indicating that the addition of sST2 did not significantly enhance risk stratification beyond NT‐proBNP. In the internal validation cohort, the combined model did not significantly improve discrimination over NT‐proBNP at 3 and 6 months (AUC: 0.774 vs. 0.706, *p* > 0.05 by DeLong test at 3 months; AUC: 0.789 vs. 0.745, *p* > 0.05 by DeLong test at 6 months; Figure 4C,D, Table 3). The improvement in C‐index was not statistically significant (ΔC‐index = 0.048, *p* > 0.05), and the NRI of 0.072 (*p* > 0.05) suggested that adding sST2 did not significantly improve risk reclassification.

### 3.7. Subgroup Analysis

Subgroup analyses based on demographic and clinical characteristics such as age, gender, BMI, and culprit artery showed that elevated sST2 correlated with higher MACE incidence (Figure 6). Interaction tests indicated no significant differences for sST2 across subgroups.

**FIGURE 6:**
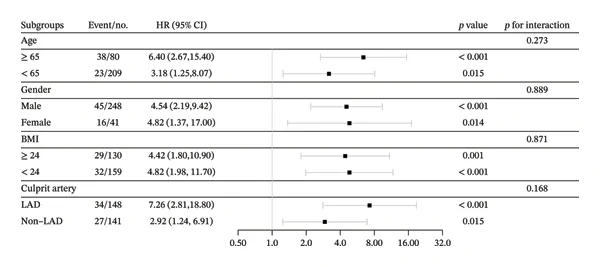
The HR of sST2 for MACE in different subgroups.

### 3.8. Component Analysis

An exploratory descriptive analysis of the individual MACE components showed that, during the 6‐month follow‐up, MACE occurred in 49 patients in the high sST2 group (68.06 per 100 person‐years) and 12 patients in the low sST2 group (16.55 per 100 person‐years), with an absolute rate difference of 51.51 per 100 person‐years (Table 4). Among the individual components, HF was the most common event in both groups (29 vs. 6), followed by recurrent MI (11 vs. 4), death (7 vs. 1), and stroke (2 vs. 1). In terms of contribution to the total absolute rate difference, HF accounted for 62.1%, followed by recurrent MI (18.9%), death (16.2%), and stroke (2.7%).

**TABLE 4:** Component‐specific event rates and contributions to the composite endpoint.

Endpoint	Events in high‐value group (*n*)	Rate per 100 person‐years in high‐value group (95% CI)	Events in low‐value group (*n*)	Rate per 100 person‐years in low‐value group (95% CI)	Absolute rate difference (high−low)	Contribution proportion (%)
MACE	49	68.06 (50.35–89.97)	12	16.55 (8.55–28.91)	51.51	100
HF	29	40.28 (26.97–57.85)	6	8.28 (3.04–18.01)	32	62.10
Recurrent MI	11	15.28 (7.63–27.34)	4	5.52 (1.50–14.13)	9.76	18.90
Death	7	9.72 (3.91–20.03)	1	1.38 (0.03–7.69)	8.34	16.20
Stroke	2	2.78 (0.34–10.03)	1	1.38 (0.03–7.69)	1.4	2.70

## 4. Discussion

By introducing the novel biomarker sST2, we observed a higher incidence of postoperative MACE in STEMI patients with elevated sST2 levels, suggesting an association between sST2 levels and MACE risk. Notably, when added to NT‐proBNP, sST2 did not significantly improve risk prediction.

The performance of sST2 may be related to its biological characteristics. As a marker of mechanical stress, sST2 participates in ventricular remodeling and inflammation. Weinberg et al. [17] reported that sST2 expression is induced after myocardial stress or injury in neonatal rat cardiomyocytes exposed to mechanical stretch. A marked increase in sST2 was detected within one hour of stimulation, which was also confirmed in cultured cardiomyocytes and in patients with acute myocardial infarction. Current evidence suggests that sST2 function is closely related to IL‐33, which inhibits angiotensin II or norepinephrine‐induced myocardial hypertrophy through NF‐κB activation [18]. Seki et al. [19] showed that IL‐33 prevents cardiomyocyte apoptosis after myocardial infarction in mice and rats, thereby improving cardiac function and survival. Similarly, Ruisong et al. [20] reported that IL‐33 attenuates myocardial ischemia–reperfusion injury after infarction. Moreover, IL‐33 interacts with a receptor complex involving ST2L, which subsequently activates NF‐κB and MAPK signaling pathways via the adaptor molecule MyD88, thereby alleviating fibrosis, inflammation, and ventricular remodeling. In contrast, sST2 acts as a competitive inhibitor by binding to IL‐33, preventing activation of the IL‐33/ST2L/IL‐1RAcP signaling complex and consequently diminishing these cardioprotective mechanisms [21]. Therefore, sST2 emerges as a possible therapeutic target to mitigate postoperative MACE among patients following myocardial infarction. According to Cunningham et al. [22], results from the PARAGON‐HF trial demonstrated that treatment with sacubitril/valsartan significantly lowered serum sST2 levels, correlating with reduced rates of hospitalization and cardiovascular mortality among HF patients exhibiting initially elevated sST2. Given that sST2 promotes myocardial hypertrophy, fibrosis, and apoptosis, regulating early sST2 expression might prevent MACE after STEMI. In this study, SGLT2i use was associated with an increased risk of MACE in univariate analysis, but this association disappeared after multivariable adjustment, possibly because patients receiving SGLT2i had a higher baseline risk (e.g., concomitant diabetes or HF). The prognostic value of sST2 cannot be attributed to SGLT2i use. At the tissue level, the study by Bergamaschi et al. [23] showed that myocardial edema and inflammation in the noninfarcted region are important histological bases of adverse remodeling after STEMI, providing a complementary perspective for sST2. However, whether the two are directly related remains to be verified by simultaneous detection in the same cohort. In addition, Fedele et al. [24] demonstrated that AF is closely associated with HF, remodeling, and inflammatory status. Although that study did not directly assess sST2, AF may influence sST2 levels by altering atrial and ventricular mechanical stress and remodeling processes; therefore, concomitant AF should be regarded as a potential confounder in the prognostic interpretation of sST2.

In this study, ROC curve analysis and C‐index calculations indicated no statistically significant difference in predictive efficacy between sST2 and NT‐proBNP across both the training and internal validation groups. Enhanced NT‐proBNP production occurs following myocardial stimulation due to ischemia, hypoxia, mechanical stretch, or neurohormonal activation, granting NT‐proBNP substantial diagnostic and prognostic significance in cardiology [25]. Evidence shows that impaired myocardial function within the infarct zone imposes increased workload on surviving cardiac tissue, causing elevated secretion of NT‐proBNP. Elevated NT‐proBNP concentrations closely reflect the degree of left ventricular dysfunction [26] and have strong predictive power regarding both short‐ and long‐term cardiovascular adverse events following myocardial infarction [27, 28]. Sabatine et al. [29] emphasized that, unlike NT‐proBNP, sST2 concentrations remain stable irrespective of age or gender, suggesting these biomarkers could play complementary roles in refining risk stratification in STEMI patients. Notably, serum sST2 is relatively unaffected by renal dysfunction [30], potentially representing a significant advantage over NT‐proBNP in myocardial infarction patients experiencing renal impairment. Furthermore, sST2 may play a more critical role in ventricular remodeling following myocardial infarction compared with NT‐proBNP [31], and it exhibits stronger correlations with adverse clinical outcomes during hospitalization [32]. However, in this cohort, sST2 did not significantly improve prediction beyond NT‐proBNP. It should therefore be regarded as a candidate biomarker warranting further investigation rather than an established tool for enhancing clinical risk assessment.

sST2 is a marker of myocardial fibrosis, and NT‐proBNP reflects ventricular wall stress. These two processes share a synergistic and complementary pathophysiological basis [33]. However, in the present study, adding sST2 to the NT‐proBNP baseline model did not significantly improve the predictive performance for MACE in STEMI patients. This null finding may be explained by several interrelated factors. First, in the early phase of STEMI, the acute elevation of ventricular wall stress triggered by myocardial ischemia and necrosis may play a dominant role, thereby partially overshadowing the early information related to fibrosis and remodeling represented by sST2. Second, myocardial fibrosis and structural remodeling are inherently progressive processes, whose clinical consequences may require longitudinal monitoring of sST2 trajectories [34] and a longer follow‐up duration to become fully apparent. Finally, as a single‐center study, the relatively limited number of events may have reduced the statistical power to detect the incremental value of the multimarker model, potentially masking its additive prognostic benefit.

Further subgroup analysis demonstrated that sST2 has some clinical utility in predicting MACE. The results showed that sST2 was significantly associated with MACE across subgroups of age, sex, BMI, and culprit vessel, and the interaction test results provided important complementary evidence supporting its clinical applicability. Consistent with our findings, previous studies have reported that sST2 levels are not correlated with traditional clinical characteristics such as age, sex, and BMI [35]. Timothy et al. [36] similarly reported no notable relationship between elevated sST2 and the location of infarction in STEMI patients. In line with this, Weir et al. [37] demonstrated no association between sST2 and infarction location, although they found significant correlations between sST2 and both infarction size and subendocardial extent, highlighting particularly elevated sST2 concentrations among patients exhibiting greater transmural infarction depth and microvascular obstruction. The application of sST2 may not require stratification threshold adjustment for specific subpopulations, which enhances its clinical applicability. Future studies are warranted to validate the generalizability of this finding in prospective multicenter cohorts.

In the component analysis, the difference in HF events accounted for 62.1% of the absolute rate difference in MACE between the two groups, suggesting that the difference in the composite endpoint may be mainly related to HF events. However, this was an exploratory descriptive analysis with a small number of events for each component, and it therefore cannot be concluded that sST2 has a component‐specific predictive effect for HF. This finding is consistent with previous studies showing that sST2 is closely associated with HF progression and adverse cardiac remodeling [38, 39]. sST2 is closely related to myocardial fibrosis and ventricular remodeling, both of which are central to the pathophysiology of HF; this mechanistic background may partly explain the relatively high proportion of HF events in the difference in the composite endpoint. From a research perspective, future studies may focus on the potential clinical value of sST2 in the postmyocardial infarction HF population and validate its value in larger samples with HF events as a prespecified endpoint.

### 4.1. Limitations

This study presents several limitations. First, this was an exploratory study. The training set included only 43 MACE events (EPV = 4.3), and although a Firth‐corrected sensitivity analysis was performed, the risk of overfitting remains and model estimates are uncertain. Performance estimates in the training set may therefore be optimistically biased. Both the training and validation sets were derived from the same single‐center dataset, constituting internal rather than external validation and limiting generalizability. Larger‐scale, multicenter studies are needed to further validate the clinical value of sST2 in predicting MACE after STEMI. Of note, sST2 measurement requires only a routine venous blood sample without additional invasive procedures, the cost per test is relatively low, and it was well accepted among enrolled patients in our center, supporting its measurement feasibility. However, a formal cost‐effectiveness analysis was not performed, which warrants further investigation. Second, postoperative dynamic serum sST2 measurements were not assessed. Future studies, as suggested by Liu et al. [40], should explore the prognostic value of dynamic sST2 variations. Lastly, the relatively short follow‐up duration of six months particularly limited our ability to observe HF events, as HF events often occur later after AMI. Therefore, this study could only evaluate the predictive value of sST2 for short‐term (6‐month) prognosis, and its findings cannot be directly extrapolated to long‐term prognosis. Future studies with longer follow‐up are needed to verify the ability of sST2 to predict long‐term prognosis.

## 5. Conclusion

STEMI continues to represent a major cardiovascular emergency, attracting ongoing clinical and research attention. Despite advancements in reperfusion techniques and medical innovations contributing to reduced hospital mortality among STEMI patients, adverse outcomes such as HF and recurrent infarction still substantially influence patient prognosis. Therefore, evaluating the risk of postoperative MACE and taking appropriate measures is crucial. In conclusion, this study showed that sST2 was associated with post‐PCI MACE in patients with STEMI and remained independently associated after adjustment for confounders, demonstrating some predictive value when used alone. However, when added to NT‐proBNP, sST2 did not significantly improve risk prediction; its incremental prognostic value was limited, and its clinical significance remains uncertain. Therefore, whether sST2 can provide clinically useful information for risk stratification in patients with STEMI independent of NT‐proBNP requires further investigation.

**Nomenclature**
ACEI: Angiotensin‐converting enzyme inhibitor
AUC: Area under the curve
BMI: Body mass index
C‐index: Concordance indices
CK–MB: Creatine kinase–myocardial band
Cr: Creatinine
CRP: C‐reactive protein
DCA: Decision curve analysis
D‐to‐W: Door to wire
HbA1C: Glycosylated hemoglobin
HF: Heart failure
Hs‐CtnI: High‐sensitivity cardiac troponin I
IL‐33: Interleukin‐33
IVS: Interventricular septal thickness
LDL‐C: Low‐density lipoprotein cholesterol
LVEDV: Left ventricular end‐diastolic volume
LVEF: Left ventricular ejection fraction
LVESV: Left ventricular end‐systolic volume
MACE: Major adverse cardiovascular events
MAPK: Mitogen‐activated protein kinases
MRA: Mineralocorticoid receptor antagonists
MYO: Myoglobin
NF‐*κ*B: Nuclear factors‐*κ*B
NRI: Net reclassification improvement
NT‐proBNP: N‐terminal pro‐brain natriuretic peptide
PCI: Percutaneous coronary intervention
RCS: Restricted cubic spline
ROC: Receiver operating characteristic
SD: Standard deviation
SGLT2i: Sodium–glucose cotransporter 2 inhibitors
sST2: Soluble suppression of tumorigenicity 2
ST2: Suppression of tumorigenicity 2
ST2L: Transmembrane form suppression of tumorigenicity 2
STEMI: Acute ST‐segment elevation myocardial infarction
TC: Total cholesterol
TG: Triglycerides
TIMI: Thrombolysis in myocardial infarction
UA: Uric acid

## Author Contributions

Jinbo Wang: conceptualization, methodology, data curation, formal analysis, and writing–original draft. Wei Du: data curation, formal analysis, and writing–original draft. Xuesong He: data curation, formal analysis, and writing–original draft. Jinglin Cheng: resources, supervision, and writing–review and editing. Zeyan Liu: methodology, resources, supervision, funding acquisition, validation, and writing–review and editing.

## Funding

The study was supported by grants from the 2024 Anhui Provincial Department of Education Key Research Project for Higher Education Institutions (Natural Sciences)—2024AH050795.

## Ethics Statement

This study was approved by the Medical Ethics Committee of the Second Affiliated Hospital of Anhui Medical University (Approval No. YX2022‐001) and conformed to the guidelines of the Declaration of Helsinki. All study participants provided written informed consent.

## Consent

Please see the Ethics Statement.

## Conflicts of Interest

The authors declare no conflicts of interest.

## Data Availability

The data that support the findings of this study are not publicly available due to privacy or ethical restrictions but are available from the corresponding author upon reasonable request.
